# The IWH-BEAT Questionnaire Validation

**DOI:** 10.3390/ijerph19063559

**Published:** 2022-03-17

**Authors:** Mario Toledo, Humberto Charles-Leija, Carlos Gustavo Castro, Iván Guerrero, Rosalinda Ballesteros-Valdés

**Affiliations:** 1 Institute for Wellbeing and Happiness, Universidad Tecmilenio, Monterrey 64909, Mexico; humbertocharles@tecmilenio.mx (H.C.-L.); cg.castro@tecmilenio.mx (C.G.C.); ivan.guerrero@tecmilenio.mx (I.G.); 2 Business School, Tecnológico de Monterrey, Monterrey 66269, Mexico

**Keywords:** organizational well-being, leadership, positive environments, meaningful work, well-being assessment

## Abstract

Positive Psychology has been devoted to enhancing well-being within organizations during the first two decades of the current millennium. Unfortunately, little data is available on current assessment related to positive psychology practices in the workplace. Therefore, to assess organizational well-being in a valid and reliable way, a new scale has been created and validated by the Institute for Wellbeing and Happiness at Tecmilenio University in Mexico: the BEAT Questionnaire whose main contribution to previous models is the element of meaningful work. EFA and CFA were carried out to determine and confirm the scale’s structure; internal consistency tests were performed too; additionally, convergence with measures of engagement, labor resources, and worker relations were also confirmed, and discriminant validity was tested by comparing associations with job search intentions and negative relationships in the workplace, yielding an instrument with four clearly defined latent dimensions, composed by 24 highly consistent items, convergent with three other valid and reliable scales. All procedures complied with statistical requirements, delivering a valid and reliable instrument for measuring well-being in the workplace.

## 1. Introduction

Promoting well-being within organizations can be seen as a necessity as well as a competitive advantage. In Mexico, from a few years to now, paying attention to the well-being of the Mexican workforce is perceived as an evident necessity. Only 13% of the Mexican workforce feels actively engaged in their work, while 60% reports feeling disconnected and 27% is actively disengaged [1]. Previous studies have shown that stress in the workplace is a worldwide chronic and urgent matter to organizations [2].

According to data from the Mexican government’s Ministry of Health [3], 75% of Mexicans suffer from fatigue due to work-related stress, which causes negative consequences at emotional, behavioral, cognitive, and physiological levels. 

The Mexican Human Resources Management Association (AMEDIRH in Spanish) reports that, annually, five thousand people fall ill due to work-related causes, and absenteeism represents a 13% loss of annual productivity; also, losses of up to three billion and three hundred thousand pesos a year are estimated due to worker’s distractions, poor time management and work-related stress [4].

Interest in individuals and organizational well-being is growing quickly. Different studies indicate that subjective well-being, life satisfaction, optimism, happiness, purpose, and other positive constructs are associated with numerous desirable outcomes, for example, lower divorce rates, higher educational and organizational success, as well as better relationships [5,6,7,8].

It has become increasingly evident that taking care of people’s well-being is an advantage for organizations. On an individual level, when workers report higher levels of well-being, they are healthier [8,9], have a higher income [10], they become promoted faster at work [11,12] and perform better [13]. According to Gallup [14], employees in the upper quartile of “engagement” increase customer satisfaction by 10%, profitability by 22% and productivity by 21%. Conversely, they report a significant decrease in turnover rates (25% in organizations with high turnover rates, 65% in organizations with low turnover rates), absenteeism (37%), and defects in service or production quality (41%). When individuals report higher levels of well-being, they show a positive influence on other people and on their communities; they demonstrate better organizational citizenship behaviors [15], inspire customer loyalty, and increase other employees’ well-being [16]. Ultimately, organizational well-being relates to profit, as organizations with higher levels of it report increases in stock market value [17] and higher earnings per share (EPS) of +147%, compared to competitors, while companies with low levels of well-being report a 2% reduction in EPS [1].

Understanding and fostering well-being should be approached from an interdisciplinary perspective at multiple levels within a system that includes individuals, organizations, communities, and nations [7].

A key element in promoting well-being is its measurement and the documentation of its changes within organizations [18,19] and even within countries [20,21]. This paper presents the psychometric development and validation, of a well-being measurement instrument: The BEAT questionnaire; some potential applications are implied too.

### 1.1. The Importance of Measuring Well-Being at Work

Unfortunately, little data is available on current assessment related to positive psychology practices in the workplace. As such, there is no critical review or study of wellness and well-being assessment practices currently available. Furthermore, as Spence [22] points out, the available data suggest that very little evaluation of organizational well-being occurs in practice, and when it does, it is usually on a superficial level.

From a measurement standpoint, operationalization of positive constructs has advanced so rapidly that their measurement in the workplace has been carried out without an adequate basis and with insufficient evidence regarding the suitability of the various instruments available to assess these constructs [23]. The implications of such a questionable measurement issue cannot be underestimated as it can result in a waste of organizational resources (time and money), inappropriate training initiatives, and in the delivery of misleading information to leaders, who in turn pass this information on to their subordinates, creating a vicious circle. It is crucial that, in the rush to harness the potential of these positive constructs, organizations and consultants (practitioners) resist the temptation to use unsupported measurement instruments or instruments that are inconsistent with the established operationalization of a construct. Rather, they must be willing to invest in the use of well-established, psychometrically supported, and widely used instruments [24].

Having said that, several benefits of using appropriate measurements in organizations could be listed, even beyond the results of the evaluations themselves. These benefits include aspects such as the perception that the organization cares about employees (e.g., improving hiring), that it is an attractive workplace (facilitating retention), that all obtained information can be used to make important management decisions (e.g., how and when to restructure a division), or that such wellness-related information can help manage both psychological and physical health in a more specific and constructive way [25,26].

### 1.2. Healthy and Resilient Organizations (HERO)

Although it is true that there is an urgent need to develop adequate methods to measure positive practices and constructs within organizations, there are important references within the practice of positive organizational psychology that serve as an example for a correct implementation of strategies of measurement in organizations. One of the strongest examples in terms of empirical evidence is the HERO model and its methodology. Moving away from Mills’ et al. Critique [24] which suggests that organizational assessments lack methodological rigor and usually present an absence of clarity in both the approach and statement of their constructs, the HERO model, clearly defines its own three constitutive elements.

The HERO model [27], refers to a combination of three main interrelated components: healthy organizational resources and practices (e.g., work resources, healthy organizational practices) as strategies to structure and organize work; healthy employees showing high levels of psychosocial well-being (e.g., confidence, work commitment); and healthy organizational results (e.g., high performance, corporate social responsibility). The HERO methodology has two main advantages related to data collection and analysis. Data is collected from different respondents at different levels of the organization (e.g., CEOs, immediate team supervisors, employees, and clients) and from objective indicators of financial performance (e.g., return on assets) using quantitative (questionnaires) and qualitative (interviews) methods. In addition, data analysis is performed at the collective stance following a multilevel perspective (that is, including individuals, teams, and organizations). Finally, it should be noted that the tool designed to evaluate this model, the HERO-Check, complies with psychometric adequacy.

When talking about measurement in formal terms, it is common for authors to use various concepts in order to name the tools they use or develop devoted to measure representative aspects of the phenomena of interest, which can be and usually are attitudes, behaviors, beliefs, preferences, degrees of agreement or disagreement about a topic, perceptions, etc.; these tools are called measuring instruments, scales, questionnaires, tests or inventories, among other various terms. For the purposes of the content of this paper, all these terms will be used synonymously with each other, therefore, if words such as scale or questionnaire are expressed, they are referring to the same concept, that is, an instrument or measurement scale: The BEAT Questionnaire.

The BEAT Questionnaire is based upon and named after an organizational well-being methodology, the BEAT Model (which is a Spanish acronym, that represents its four main elements: Organizational well-being, Leader’s approach, Positive environments, and Meaningful work) developed by the Institute for Wellbeing and Happiness (IWH) in Tecmilenio University, México. Although the acronym was conceived in and it is ideal for reading in Spanish, the English meaning of the word BEAT also refers to the pulse, in other words, what brings life to an organization; it is due to this coincidence that the authors believe it works in both contexts.

A common difficulty researchers and practitioners often deal with these types of evaluations is that management often agrees for this kind of studies to be undertaken only at an employee level, but not at management level itself. Following the example of already validated scales such as the Hero-Check [27] the BEAT questionnaire seeks to evaluate its constructs at different levels of the organization, focusing on managers and supervisors as well as employees. 

Despite being a quantitative measurement tool, it can be added to comprehensive evaluation processes that seek evidence of the organization’s resources (facilities, HR management programs, etc.) in a qualitative way. Another benefit is the briefness in its extension and application time, which can be less than 10 min, which stimulates the response rate since it is less tiring for the participants, compared to what has been seen in exhaustive evaluations [28]. In addition, it seeks to comply with adequate psychometric thoroughness, that is, to achieve measurement validity and reliability. 

### 1.3. The BEAT Scale: The Pulse of the Organization

#### 1.3.1. Organizational Well-Being

One element that all work environments have in common is the relationship between labor requirements and job resources. Labor requirements are those physical, psychological, organizational, or social aspects of work that demand sustained effort and have a physiological and psychological toll on workers [29]. Some harmful examples of these demands can be work overload, as well as routine or role conflict. On the other hand, work resources are those physical, psychological, or organizational aspects that serve up to achieve objectives, reduce the harmful effects of work requirements, and stimulate personal growth [30].

When there is an imbalance between labor requirements and resources, a process of erosion is generated so that it is reflected in symptoms of organizational-derived disease, such as “burnout”, while when requirements and resources are well balanced, a process of motivation is usually generated and reflected in positive states related to well-being, such as “engagement” [27,28,29,30,31]. 

One of the elements proposed by organizational well-being models is the implementation of resources and organizational practices that help employees to be able to overcome work challenges daily. Based on the elements proposed in the HERO model, some positive practices can be named, such as work-life balance, mobbing prevention, psychosocial health, career development, and organizational communication. On the other hand, task resources that enable the performance of collaborators should also be included, for example, autonomy, feedback, teamwork, and the promotion of positive and transformational leadership [32].

The BEAT questionnaire allows workers to have a voice: to indicate what is good or bad within the organization, and what they perceive the company offers them in order to increase the chances of flourishing as people and professionals, which is in line with the approaches of Cameron et al., [33]. The worker can express if he or she considers that the organization conveys a sincere concern for his or her well-being, as well as his or her perception of justice during hiring and promotion processes. It is valuable that workers perceive that their effort and loyalty are deemed, since a worker who does not perceive that his or her company values his or her work, will have less commitment and will be less dedicated in their tasks within the workplace. The well-being of an organization should not be seen as an individual responsibility but as a shared one; an organization that provides sufficient resources to its collaborators will have a greater opportunity to impact their well-being and its own flourishing.

#### 1.3.2. Focus on Positive Leadership

It is said that people do not quit their job, they quit their leader. Leadership is increasingly becoming an important research topic, and different leadership styles play an important role in promoting employee well-being and organization results [34,35].

Positive leadership is based on the application of positive principles of behavior that emerged from Positive Organizational Psychology also known as POP [36]. Specifically, positive leadership has three basic components: it focuses on those people’s strengths and abilities that reaffirm their human potential, emphasizes results, and facilitates above-average individual and organizational performance; its field of action focuses on those components that can be conceived as essential virtues of the human condition. According to Cameron [34] there are four ways or strategies for the implementation of positive leadership: fostering positive emotions such as compassion, forgiveness, and gratitude; creating relational energy as a result of positive relationships; using supportive communication in positive feedback processes; and doing a positive, meaningful work [33,34,37,38]. 

Thus, the BEAT questionnaire allows the collaborator to evaluate the approach that his or her leader has, based on the theoretical framework provided by the positive leadership approach.

#### 1.3.3. Positive Environmental Features

Relationships in the workplace are fundamental for personal well-being, but also for productivity. People relate to each other at work and build ties, and given the human natural need for interaction and connection, having a positive environment where people can develop positive and meaningful relationships at work is an important factor to consider while improving wellbeing at the workplace. Studies such as Raile et al. [39] found that strong connections (i.e., friendship at work) showed a positive correlation with psychological wellbeing and job satisfaction. In addition, positive relationships can be an important source of relational energy; Bakker et al. [40] found that teams with a higher density network report a greater level of energy and performance. Dutton [41], suggest that there are practical strategies for building high-quality connections at work, such as respectful engagement, task enabling, and trust.

Positive interactions play an important role in the workplace, specially predicting team performance. Following on Gottman’s [42] research on married couples, Losada et al. [43] found that the same ratio of positive to negative interactions (5 to 1) is the critical differentiator between high-, medium-, and low-performing teams.

Fostering a shared identity in the workplace is another important feature for creating a positive environment. In a study conducted by Sherif [44], introducing compellingly shared goals that required the collaborative efforts of all, proved to be an effective strategy for reducing tension between team members. The common in-group identity model can explain this effect, when people are induced to recategorize themselves as a superordinate group rather than as separate groups, out-group biases are reduced (i.e., prejudice, discrimination). This has huge implications for building a more positive environment where people can thrive in their workplace. 

#### 1.3.4. Meaningful Work

Psychology of working theory (PWT) explains how structural and psychological factors have an impact on accessing a fulfilling job [45]. According to this theory, one of its key elements is Meaningful work. Steger et al. [46] define meaningful work by three key elements; positive meaning refers to a job that has a personal significance; meaning making trough work, which im-plies that people’s work can help them build meaning in their own lives; and greater good motivation, a facet that reflects commonly held ideas that work is most meaningful when it has a broader impact on others.

Psychology of working theory (PWT) explains how structural and psychological factors have an impact on accessing a fulfilling job [45]. According to this theory, one of its key elements is Meaningful work. Steger et al. [46] define meaningful work by three key elements; positive meaning, refers to a job that has a personal significance; meaning making trough work, which implies that people’s work can help them build meaning in their own lives; and greater good motivation, a facet that reflects commonly held ideas that work is most meaningful when it has a broader impact on others.

Meaningfulness is a fundamental human and psychological need; when satisfied, it can lead to positives outcomes, such as work engagement, job satisfaction, life satisfaction, life expectancy, mental and physical health, wellbeing, meaning in life, organizational citizenship behaviors, self-rated job performance, and withdrawal intentions [47,48,49,50,51]; when unsatisfied, it can lead to negative outcomes, such as substance abuse, suicidal ideation, alienation, and poor workplace performance [50].

PWT suggests that access to a meaningful and fulfilling work is attributable to decent work (i.e., fair income, non-discriminatory) via three groups of basic needs: (a) survival/power needs, which refer to basic needs such as food and shelter, and access to opportunities; (b) social contribution needs, which refer to the need to contribute and belonging, and (c) self-determination needs, which include autonomy, competence, and relatedness [45]. In other words, work can be a pathway not only to meet basic needs, but to also, to fulfill deeper ones such as belonging to a community and integrating intrinsic values into action.

People want their careers and their work to be more than simply a way to earn a paycheck; they want their work to mean something [49]. For example, when people´s tasks contribute to the well-being of others, they are much more rewarding, meaningful, and transcendental [52,53].

The BEAT questionnaire is designed to offer the collaborator a possibility of evaluating whether their work is meaningful for them, if their tasks contribute to a better understanding of their world, make sense of the world around them, and contribute to a greater good.

#### 1.3.5. Contributions of the BEAT Scale

The BEAT scale integrates different elements related to well-being within organizations. It picks up elements from other positive organization models such as the organizational resources of the HERO model, the positive leader strategies of Cameron [34], and the high-quality connections of Dutton [41]. However, its greatest contribution to the construction of a model that evaluates such constitutive factors of a positive organization is the element of meaningful work.

The search for meaning is a human need for most people, and work is one of the main activities that contribute to the satisfaction of that need. By allowing both the expression of individuality and the opportunity to make a purposeful contribution while connecting with others [52], work can help with meeting that need. While models such as HERO consider social and organizational resources, as well as leadership as elements that constitute a positive organization, the BEAT model also proposes the evaluation of, to what extent employees perceive that they contribute to society or that their work helps them satisfy their need for significance.

When people pursue a deep purpose or become involved in a work that is personally important, they experience significant positive effects, such as: higher levels of engagement, empowerment, satisfaction, and a sense of accomplishment [53,54]. As described in the literature, it is because of such effects of meaningful work on people that it is considered a capital element for the constitution of a positive organization.

## 2. Materials and Methods

### 2.1. Participants

For the present study, data of 395 employees of Tecmilenio, a private university in Mexico, were collected during the month of April 2021. 257 women (65.1%) and 135 men (34.2%) were surveyed, while 3 people (0.8%) preferred not to specify their gender. Seven university campuses in different states of the Mexican Republic participated: Culiacan (*n* = 38), Ferrería (*n* = 67), Laguna (*n* = 29), Las Torres, (*n* = 123), Mazatlán (*n* = 44), San Luis Potosí (*n* = 56), and Veracruz (*n* = 38). Initially, data of 474 collaborators were collected, but in the end, only 395 complete surveys were kept for the analysis.

### 2.2. Measurements

The BEAT questionnaire is made up of 24 items that evaluate the four elements of the model: *Organizational Well-being* (5 items), *Positive leadership* (8 items), *Positive environments* (3 items) and *Meaningful work* (8 items). It uses a Likert scale ranging from 1 to 5 where 1 is totally disagree and 5 is totally agree.

To analyze the convergent validity of the BEAT questionnaire, correlations with other elements of well-being in organizations were sought, such as engagement, satisfaction with labor resources, and discriminant validity with worker relations and job search. A variety of studies showed a positive correlation between the BEAT elements with engagement, and satisfaction with organizational and leadership resources [55,56,57]; and a negative correlation with poor work relations [58] and job search [59].

For the aims of this study, the adaptation of the UWES-9 to the Mexican population composed by 9 items was used [28]. The UWES-9 measure three components of engagement: vigor, absorption, and dedication.

The scale of satisfaction with work resources (CSRL-16) [57] based upon the HERO model [27] has 16 items that measure people’s satisfaction with organizational resources: *Resources of the leader* (4 items in CSRLL), *Task resources* (4 items in CSRLT), *Organizational resources* (4 items in CSRLO).

The worker relations scale (WRS) [60] is a measure used to test discriminant validity. The three-question scale measures unsatisfactory labor relations. It is expected to show a negative correlation with all the constructs evaluated in the BEAT questionnaire.

A second scale to measure discriminant validity was the job search (JS) question, adapted from Bluedorn [59]. Companies with high levels on the BEAT scale are expected to have fewer employees seeking other job opportunities.

### 2.3. Procedure

To perform the psychometric validation of the BEAT scale, data from seven different campuses were collected. Face validity was obtained through the opinions of the people from HR departments who agreed and gave approval to the questionnaire. The questionnaire was launched through an electronic link (using the Qualtrics platform) and disseminated via an email that included an invitation to participate along with a detailed description of the research objectives and instructions for answering the questionnaire. To address ethical issues, the procedures complied with the ethical standards in the Mexican Ethical Code of the Psychologist [61]. Participation was voluntary and informed consent was obtained after the question: “Do you agree to participate in this research by answering the survey?” The participants were asked to click on a yes or no box, after which they could complete the questionnaire; if they refused (by answering no), the survey was automatically canceled. Anonymity and confidentiality were always guaranteed. All procedures were non-invasive, and no harm was induced as a consequence of participation in the study.

### 2.4. Analytical Procedure

The psychometric analysis of the BEAT scale consisted in (a) exploratory factorial analysis (EFA) to examine the theoretical structure of the instrument, (b) internal consistency tests, (c) convergent and discriminant validity tests and finally, (d) confirmatory factorial analysis (CFA) to test the robustness of the model.

Initially, an exploratory factor analysis (EFA) with all 43 items of the instrument was performed and the obtained matrix showed that some items loaded poorly with respect to factors; due to this reason, it was decided to eliminate them from subsequent analyzes; following rounds of EFA and internal consistency analyses were performed yielding a final four factor solution with 24 items.

Later, the original sample of 395 individuals was randomly split to generate two different datasets (N1 = 199 and N2 = 196); 199 observations were used for a new exploratory factor analysis (EFA) and internal consistency tests to re-examine the theoretical structure of the instrument with 24 items; in addition, with the aim of investigating whether the factor structure can be replicated in the new dataset from 196 participants (N2), a confirmatory factor analysis (CFA) was conducted.

Additionally, in order to test construct validity, the composite reliability coefficient (CR), the average variance extracted (AVE) and their respective standardized factor loadings along with its standardized error were obtained. Correlations (Pearson r) with CSRL, UWES-9 as proxy criterion measures and the WRS scale and a Job Search (JS) item as discriminant measures.

These final EFA, internal consistency analysis CFA, and correlations were completed with 24 items and the results are presented in the next section. Data were analyzed using SPSS (21), Excel (2019) and R (3.6).

## 3. Results

### 3.1. Exploratory Factor Analysis

Concerning the construct validity, an exploratory factor analysis (EFA) with factor extraction by principal components analysis (PCA) was performed to reduce the number of variables and detect redundant items or items that provide little information [62]. It was explored if the items explained a great amount of the common variance or if they rather reflected uniqueness. The EFA showed a balanced four factors result with a common variance within factor-items and also parallel or tau-equivalent items composing each one.

These factors were expected to be the four constructs of the BEAT Model a priori, yet this analysis was performed in an exploratory fashion and, to confirm the BEAT model scale structure, a confirmatory factor analysis (CFA) was carried on too, as shown in the following pages.

To assess the strength of the relationship between pairs of variables or items based on partial correlations, and to verify that the number of explanatory factors is small, the Kaiser-Meyer-Olkin (KMO) test was used. The value obtained was 0.945, which justifies a factor analysis [62]. Regarding Bartlett’s Test of Sphericity, it is shown that, in fact, there is a statistically significant correlation between the variables (*p* < 0.05.). Therefore, the factor analysis is applicable, as shown in Table 1.

Principal components with eigenvalues greater than 1 were considered for the EFA, as recommended by specialized literature (see eigenvalue rule, Kaiser) [62,63,64]. Data show that there are four principal components to be considered. These factors explain 62.4% of the variance, as shown in Table 1. Table 2 shows both item distribution through all four factors (or principal components) and the number of items composing each one of them, as well as the factor loadings which ranged from 0.595 to 0.878.

#### 3.1.1. Reliability and Item Analysis

To determine the reliability of the instrument, Cronbach’s alpha values were obtained for each latent dimension of the scale. All Cronbach’s values are higher than 0.80, which is an indicator of high internal consistency or equivalence between parallel items of each latent factor or dimension [65]. The instrument obtained a global alpha close to 0.95, which is considered a coefficient that acceptably represents the magnitude of the correlation between all 24 items, as shown in Table 1.

#### 3.1.2. Convergent and Discriminant Validity

Table 3 shows latent dimensions (factors) with their respective standardized factor loadings, its composite reliability (CR) and the average variance extracted (AVE). As can be seen, the CR and AVE coefficients were above the minimum criteria of CR ≥ 0.7 and AVE ≥ 0.5 [66].

The Table 4 shows correlations between the items that make up the four factors of the BEAT questionnaire compared with the CSRL proposed by Spontón, Trógolo, Castellano, Morera, and Medrano [57]. Correlation between the UWES-9 engagement scale, Spanish version of Hernandez-Vargas et al. [28], and the BEAT scale are also observed. Finally, there is a negative correlation between BEAT JS and WRS, as expected, because items of WRS measure negative relationship with co-workers and JS measures the intention of finding a new job, deemed as job search.

### 3.2. Confirmatory Factor Analysis

In order to verify the BEAT scale measurement model, a confirmatory factor analysis (CFA), which is a particular case of a structural equation model (SEM), was carried out. The model was specified with each variable by saturating only on the common factor that it measures, and the unique factors are uncorrelated (although they covary together). The CFA was carried out using the maximum likelihood (ML) estimator according to the assumption of multivariate normality of the items [67] and the nonlinear minimization subject to box constraints (NLMINB) optimization method, to avoid false convergence.

**Figure 1:**
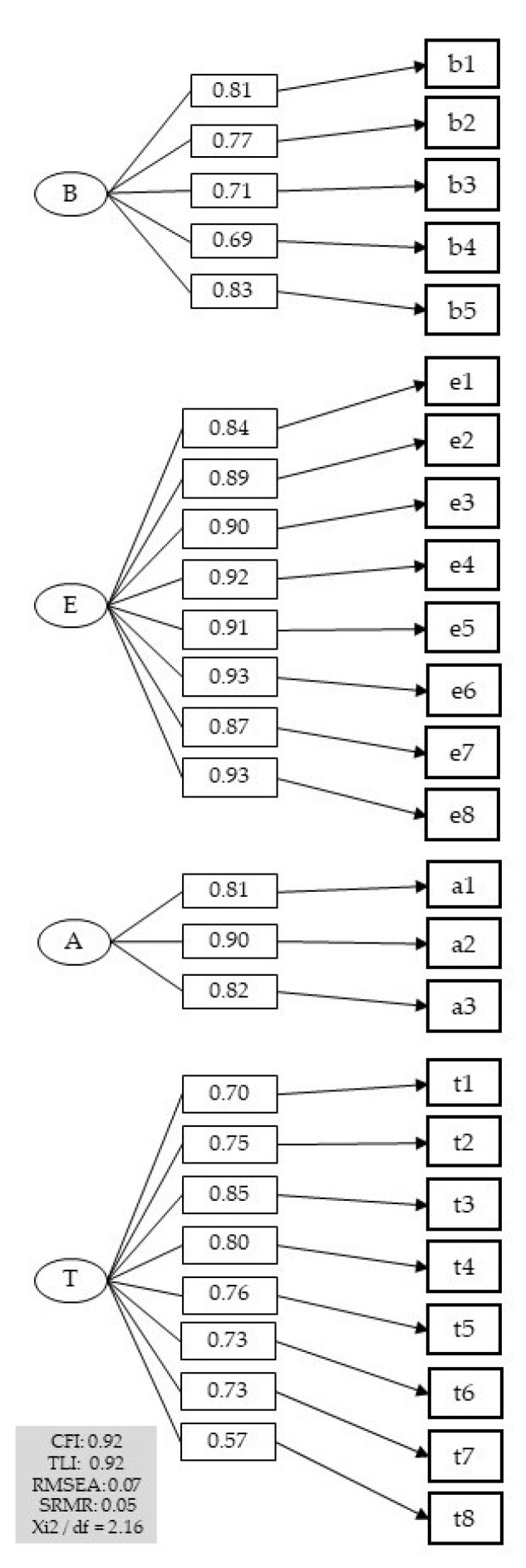
BEAT Questionnaire CFA Model. Note: *p* = 0.000. B: Organizational Well-Being. E: Focus in Leadership. A: Positive Environments. T: Meaningful Work.

Research literature recommends a comparative fit index (CFI) and a Tucker and Lewis index (TLI) greater than 0.90, as well as a mean square error of approximation (RMSEA) less than 0.06 ideally and less than 0.08 [68] acceptably a standardized root mean square residual (SRMR) less than 0.08 [69]. Regarding the absolute and relative indexes of the model, the current analysis yielded all these coefficients (N = 196; TLI = 0.920; CFI = 0.928; RMSEA = 0.077; SRMR = 0.054; Xi^2^/df = 2.16). see Figure 1.

The graphical model shows the four latent variables clearly separated and the items belonging to and determined by each one. Factor loadings are robust and significant.

## 4. Discussion

After an exhaustive process, an instrument was developed to evaluate four elements that constitute a positive organization (Wellbeing resources, Leadership, Positive environments, and Meaningful work): the BEAT Questionnaire, in correspondence with its Spanish acronym.

Validity and reliability analyses of the BEAT scale showed that this is an instrument with adequate psychometric properties for measuring the proposed variables of interest. On the one hand, exploratory factor analyses (EFA) revealed an adequate structure representing the theoretical model. When rotating the matrix, the four BEAT dimensions were clearly observed; furthermore, factor loadings of the preserved items after preliminary analysis were appropriate. On the other hand, an internal consistency analysis (testing reliability) complemented EFA findings by showing that all Cronbach’s values displayed high internal consistency or equivalence between parallel items of each latent factor or dimension; the instrument obtained a global alpha that acceptably represents the magnitude of the correlation between all 24 items.

In addition, the CR and AVE indicators evaluated the construct validity appropriately and findings derived from correlations with UWES-9 and CSRL, performed for testing convergent validity, exposed that organizational well-being with organizational resources, focus on positive leadership with leader resources, positive environments with team relationships, and meaningful work with task resources were highly correlated. The convergence with these three scales reflects that the BEAT well-being scale represents an adequate measurement of the well-being that employees experience in organizations. On the other hand, WRS and JS showed the negative correlation expected.

Confirmatory modelling of data using CFA also showed that (a) the instrument measures the four BEAT dimensions adequately and (b) all items are grouped with appropriate factor loadings. Regarding the absolute and relative indexes of the models, current analyses yielded reasonably acceptable coefficients. The RMSEA was at an acceptable but not excellent level as proposed by authors [70]; however, the other indexes showed better adequacy, implying an overall goodness of fit for both models (the correlations’ path and the covariances’ path models).

Although there is recent objective criticism towards the methods of the classical test theory, also known as true score theory, when it measures reliability and the exploratory standpoint represented by EFA methods when it measures validity [67], the large psychometric tradition has been based upon these methods to such an extent that they have become standard practices [71,72,73]. Therefore, the authors of this article have preferred to take advantage of both positions and their benefits in a complementary sense, recognizing that the debate remains open.

In general terms, using both approaches (those derived from classical measurement theories as well as those developments derived from the a priori perspective embodied by precursors of SEM modeling such as CFA) showed that the scale meets the requirements prescribed in classic psychometric development studies [71,72,73,74] as well as in recent ones devoted to testing each item’s validity and reliability [67]. Altogether these procedures render empirical evidence that the instrument has adequate psychometric properties and confirms its validity and reliability.

One benefit that can be obtained by using the BEAT scale is the possibility of evaluating multiple spheres. When reporting BEAT scale results to organizations, it is highly recommended that the multidimensional structure be kept and interpreted, instead of condensing a unique score. While a single overall score could provide an overview of organizational well-being, such an approximation will disguise the variation between its different composing elements. For example, presenting an overall score to a company may be insufficient when conducting an intervention. Instead, if any given organization scores low in meaningful work, interventions can aim to develop strategies such as “job crafting” to build a more meaningful perception of work rather than trying to fix the other organizational well-being aspects that might be working good otherwise [56,75].

### Limitations

It is not possible to recommend an ideal scoring profile for the BEAT scale because cut points haven’t been established yet due to sampling size limitations. There is a lack of representativeness of the sample because the study was carried out in only one university (even though it has campuses across the Country) with workers who might already be trained in a previously long-established work well-being culture, producing a ceiling effect from the start. Consequently, the instrument is at a descriptive stage, but is still useful to obtain valuable information when comparing group performances to a certain but reliable extent.

There is still a need to validate the tool in wider samples to achieve adequate representativeness; test-retest and cross validation procedures are still required too. In addition, based upon different contextual aspects and realities of the companies, diverse score profiles are expected depending on their type, size, or line of business. Currently, data from 32 companies all over the Mexican territory are being processed and analyzed with the aim of improving the instrument’s general applicability as a potential decision-making tool for the industry. Future research should examine contextual variations that may influence the BEAT questionnaire scoring.

## 5. Conclusions

In conclusion, it can be stressed that the BEAT questionnaire is an instrument with adequate psychometric properties for measuring paramount, constitutive organizational well-being aspects in line to theoretical proposals such as the HERO model and the BEAT model developed by the IWH of Tecmilenio University.

*Organizational Well-being*. The BEAT scale can be a useful tool to apply in organizations as it gives employees a voice by allowing them to talk about how they feel regarding the resources that their organization provides them with to improve their well-being. By knowing this information, companies can play an active role in the development of motivational processes that lead to higher levels of work commitment and, naturally, well-being.

*Focus on leadership.* Assessing the leader’s approach is also a useful strategy for the sake of achieving higher well-being levels within companies. Different studies reaffirm the impact that leaders have on their collaborators’ well-being and the organization’s productivity and results. The promotion of forgiveness, compassion, and gratitude, as sources of relational energy and supportive communication, create greater well-being, performance, and are paramount features to be measured too.

*Positive environments*. Research carried out by POP has confirmed that relationships are a great predictor of well-being both at work and in life. For companies, it is capital to rely on valid measures of how relationships are lived within the organization. The BEAT scale can offer key information features to guide the decision-making process to help organization and teams develop better personnel interactions, greater group cohesion, support, and commitment.

*Meaningful work*. In addition to evaluating those elements that literature points out as factors of well-being in organizations, the BEAT questionnaire measures the meaning employees give to their work, enabling a way to know more about their perception about daily tasks, and the impact of these tasks on the employee’s well-being.

In future research, the authors suggest the possibility of analyzing factual relations between the BEAT model and other positive organizational behaviors such as engagement, productivity, job satisfaction as well as negative outcome variables such as burnout, turnover rates, etc.

The BEAT model, based on recent findings from the POP, offers the possibility for organizations to know themselves better and identify areas of opportunity related to institutional well-being. In addition, providing employees with the opportunity of increasing their own well-being is an idea that is gradually gaining presence in organizations’ cultural change, and the tool presented in this article is an effort to have an instrument that provides useful, valid, and replicable information.

## Figures and Tables

**Table 1:** Eigenvalues, variance, and Cronbach’s α.

	B	E	A	T
Eigenvalues	9.75	3.37	1.83	1.49
Variance accounted for each factor %	40.61	14.06	7.61	6.20
Cumulative variance accounted for each solution (%)	40.61	54.68	62.28	68.48
α	0.79	0.96	0.89	0.89
N of Items	5	8	3	8

BEAT Scale’s α = 0.93, KMO = 0.92, Approx. Chi-Square 3513.35, df = 276, Total of items = 24.

**Table 2:** Factorial loadings after varimax rotation.

	B	E	A	T	Communalities
b1	0.10	0.11	**0.76**	0.04	0.59
b2	0.09	0.17	**0.70**	0.03	0.53
b3	0.06	0.10	**0.73**	0.15	0.58
b4	0.12	0.27	**0.58**	0.19	0.46
b5	0.24	0.27	**0.68**	0.13	0.61
e1	**0.83**	0.13	0.05	0.14	0.73
e2	**0.90**	0.16	0.09	0.12	0.86
e3	**0.83**	0.25	0.11	0.15	0.78
e4	**0.86**	0.19	0.15	0.15	0.83
e5	**0.88**	0.15	0.17	0.13	0.83
e6	**0.86**	0.15	0.15	0.09	0.79
e7	**0.87**	0.12	0.14	0.19	0.83
e8	**0.85**	0.10	0.09	0.23	0.79
a1	0.20	0.12	0.17	**0.86**	0.82
a2	0.32	0.21	0.14	**0.83**	0.85
a3	0.28	0.20	0.17	**0.79**	0.77
t1	0.21	**0.72**	0.05	0.01	0.57
t2	0.00	**0.75**	0.11	0.02	0.58
t3	0.17	**0.74**	0.14	0.09	0.61
t4	0.05	**0.83**	0.09	0.08	0.70
t5	0.20	**0.78**	0.24	0.11	0.72
t6	0.16	**0.71**	0.26	0.18	0.63
t7	0.27	**0.64**	0.31	0.19	0.62
t8	0.19	**0.51**	0.14	0.25	0.38

Extraction method: Principal components analysis. Rotation method: Varimax with Kaiser normalization. The rotation converged in 7 iterations. Bold font indicates that the factor loading is significant at 95% level.

**Table 3:** Overall Confirmatory Factor Analysis (CFA) for the BEAT Measurement Model.

Constructs and Items			Factor Loadings	SE	CR	AVE
**B**		psycho-social health	0.81 0.71 0.83	0.04 0.05 0.05		
	Healthy Organization Practices	Information and communication	0.77	0.04	0.87	0.58
		Skill development	0.69	0.05		
**E**		Positive communication	0.84 0.89 0.9	0.03 0.02 0.01		
	Positive Leadership	Strength recognition	0.92 0.91	0.01 0.01	0.97	0.81
		Positive relationships	0.93 0.87 0.93	0.01 0.02 0.01		
**A**		Task enabling	0.81	0.02		
	Positive Environments	Trust	0.90	0.02	0.88	0.71
		Respectful engagement	0.82	0.02		
**T**		Positive meaning	0.76 0.73 0.73	0.01 0.03 0.02		
	Meaningful Work	Meaning Making	0.70 0.85 0.57	0.01 0.01 0.01	0.91	0.55
		Greater good motivation	0.75 0.80	0.01 0.01		

**Table 4:** Convergent and discriminant validity (BEAT, CSRL, UWES, WRS and JS).

	BEAT	B	E	A	T	CSRLO	CSRL	CSRL	WRS	UWES-9	JS
BEAT	1										
B	0.670 **	1									
E	0.859 **	0.350 **	1								
A	0.686 **	0.394 **	0.489 **	1							
T	0.743 **	0.491 **	0.416 **	0.419 **	1						
CSRLO	0.656 **	0.552 **	0.457 **	0.460 **	0.568 **	1					
CSRLL	0.745 **	0.334 **	0.851 **	0.422 **	0.357 **	0.480 **	1				
CSRLT	0.679 **	0.588 **	0.438 **	0.474 **	0.639 **	0.680 **	0.488 **	1			
WRS	−0.477 **	−0.348 **	−0.333 **	−0.572 **	−0.312 **	−0.356 **	−0.259 **	−0.355 **	1		
UWES-9	0.587 **	0.404 **	0.361 **	0.390 **	0.678 **	0.567 **	0.343 **	0.665 **	−0.366 **	1	
JS	−0.301 **	−0.142 *	−0.324 **	−0.129	−0.194 **	−0.417 **	−0.321 **	−0.292 **	0.100	−0.214 **	1

**. Correlation is significant at *p* < 0.01 (two-tailed test), *. Correlation is significant at *p* < 0.05 (two-tailed test).

## Data Availability

The questionnaire can be available upon request only for research, non-commercial purposes. To access the analytic information, please refer to https://drive.google.com/drive/folders/1NbEQbkwYegs73oiFlKq9P01NFAs5KL8q?usp=sharing (accessed on 13 March 2022).
